# Structural, Binding and Functional Properties of Milk Protein-Polyphenol Systems: A Review

**DOI:** 10.3390/molecules28052288

**Published:** 2023-03-01

**Authors:** Tessa M. van de Langerijt, James A. O’Mahony, Shane V. Crowley

**Affiliations:** School of Food and Nutritional Sciences, University College Cork, T12 YN60 Cork, Ireland

**Keywords:** polyphenols, milk proteins, micelles, aggregates, blood serum albumin aggregates, native casein micelles, re-assembled casein micelles, casein nanoparticles, complex formation, protein-polyphenol interaction

## Abstract

Polyphenols (PP) are linked to health benefits (e.g., prevention of cancer, cardiovascular disease and obesity), which are mainly attributed to their antioxidant activity. During digestion, PP are oxidised to a significant degree reducing their bio-functionality. In recent years, the potential of various milk protein systems, including β-casein micelles, β-lactoglobulin aggregates, blood serum albumin aggregates, native casein micelles and re-assembled casein micelles, to bind and protect PP have been investigated. These studies have yet to be systematically reviewed. The functional properties of the milk protein-PP systems depend on the type and concentration of both PP and protein, as well as the structure of the resultant complexes, with environmental and processing factors also having an influence. Milk protein systems protect PP from degradation during digestion, resulting in a higher bioaccessibility and bioavailability, which improve the functional properties of PP upon consumption. This review compares different milk protein systems in terms of physicochemical properties, PP binding performance and ability to enhance the bio-functional properties of PP. The goal is to provide a comprehensive overview on the structural, binding, and functional properties of milk protein-polyphenol systems. It is concluded that milk protein complexes function effectively as delivery systems for PP, protecting PP from oxidation during digestion.

## 1. Introduction

**Figure 1:**
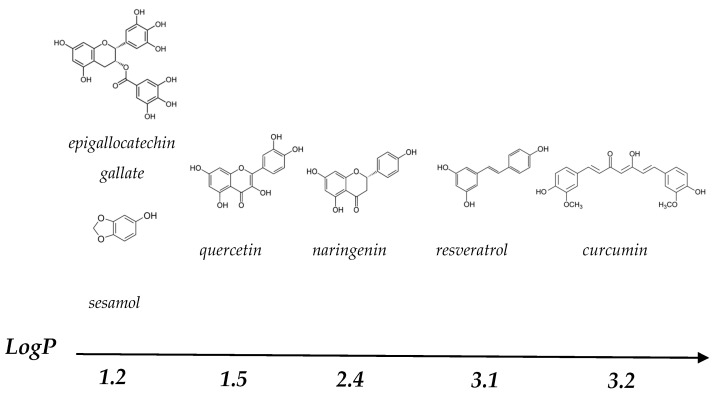
Molecular structure of different polyphenols ordered from left to right in terms of their decreasing polarity, expressed as LogP, which values were calculated by XLogP3 3.0 (PubChem release 7 May 2021).

Polyphenols (PP) are secondary plant metabolites containing an aromatic benzenoid ring and a hydroxyl group [1]. There is a great variety of PP, with estimates of approximately 8000–10,000 different compounds, with sizes ranging from simple monomers to complex polymers [2,3]. The structures of the PP most commonly studied in the literature are summarised in Figure 1.

PP have many different biological functions in plants, including pigmentation, growth and reproduction [4]. In recent years, consumption of PP has been linked to positive health effects, due to their antioxidant properties, which protects cells from oxidative damage, and positive effects on the gut microbiota. This could potentially reduce the risk of certain diseases, e.g., cancer, cardiovascular disease and inflammation [5,6], making PP of considerable interest as functional ingredients in the food industry. However, many PP have low solubility in water, because of their nonpolar aromatic benzenoid rings [7]. They are also sensitive to oxidation during digestion, due to the slightly alkaline conditions of the small intestine, which leads to a decrease in bioaccessibility and lower bioavailability [8,9]. Binding of PP by milk proteins could potentially improve their water solubility and limit oxidation during digestion [10,11,12]. 

Bovine milk contains ~3.3% (*w*/*w*) protein, which consists of more than 100 different types of proteins, with the two main families being caseins (80%) and whey protein (20%) [13]. Caseins consist of four major different types, α_s1_-, α_s2_-, β- and κ-casein, which represent 38, 10, 35 and 12% of the caseins, respectively [14]. They are mostly present in colloidal aggregates, called casein micelles (CMs), and have a high concentration of calcium phosphate [15]. The CMs have an average diameter of 150–180 nm and average molecular weight of 10^8^ kDa [16,17]. There are multiple models describing the structure of CMs, but in general it is believed that α_s1_-, α_s2_- and β-casein form loose aggregates through hydrophobic interactions, which are stabilised by interactions between colloidal calcium phosphate (CCP) and casein phosphoserine. κ-Casein forms a hairy layer at the surface of the CM, providing steric and electrostatic stabilisation [18,19,20]. Water channels run through this structure, which makes it possible for small molecules, such as PP, to penetrate into the core of the CMs [20,21].

The main types of whey proteins are β-lactoglobulin (β-Lg), α-lactalbumin (α-La), blood serum albumin (BSA) and immunoglobulins (Igs), which represent ~60, 20, 10 and 10% of total whey protein, respectively [14]. Whey proteins are globular proteins and have a high degree of secondary structure. Heating of these proteins above their denaturation temperature (which range between ~60–80 °C), will lead to partial unfolding. This causes the exposure of hydrophobic groups that were previously buried within the inside of the structure, which can result in aggregation [22] to an extent depending on environmental conditions including temperature, pH and ionic strength [23].

There is a great variety of milk protein types and structures, depending on the origin and processing history of the milk. Individual proteins or groups of protein can be separated by membrane filtration or ion exchange chromatography, generating concentrates, isolates and fractions, such as milk protein concentrate and isolate. Other milk protein products are produced by acidification, enzyme treatments and/or salt addition, including acid casein, caseinates and rennet casein [14]. It is uncertain how structural differences between such milk protein-rich ingredients, associated with processing history, may affect binding to PP.

The main interactions between milk proteins and PP are non-covalent bonds consisting of hydrophobic and hydrophilic interactions, which include hydrogen bonds, van der Waals and electrostatic interactions [24,25]:

- Hydrophobic interactions occur between the aromatic benzenoid rings of PP and the hydrophobic regions of proteins. These interactions are temperature-dependent and a reduction in temperature decreases the strength of the hydrophobic interaction between proteins and PP.
- A hydrogen bond is a dipole-dipole interaction, involving hydrogen and electronegative ions, e.g., Cl, F, N, O and S. The hydrogen bonds between proteins and PP are mainly between the hydroxyl groups of the PP and the polar amino acids of the proteins, and an increase in temperature will lead to a decrease in the amount of hydrogen bonds.
- Van der Waals interactions are the attractive forces between dipoles of non-charged atoms and molecules and the distance between the dipoles determines the strength of the van der Waals interactions. Temperature has a small influence on the strength of van der Waals interactions.
- Electrostatic interactions are the interactions between two charged atoms or molecules. Depending on pH, the amino acid residues of proteins can be positively charged (arginine, histidine and lysine) or negatively charged (aspartic acid and glutamic acid). These amino acids can interact with PP of an opposite charge through electrostatic interactions. In addition, salt ions can screen the charges present on the protein and PP, which will lead to decreased electrostatic interactions.

**Figure 2:**
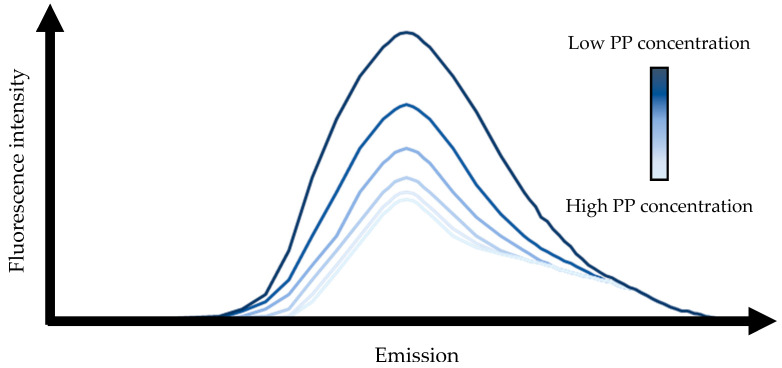
Schematic representation of a fluorescence emission spectra, in which the fluorescence emission decreases with an increasing polyphenol (PP) concentration.

PP-protein binding is often studied by fluorescence spectroscopy [21,26,27], with the amino acids tryptophan, tyrosine and phenylalanine having a fluorescence emission. After excitation the systems fluorescence emission can be measured, which is typically completed for systems with the same protein concentration and an increasing concentration of PP. When the fluorescent amino acids interact with PP, fluorescence intensity quenching follows, which results in a decrease in fluorescence emission upon an increase of the PP concentration (Figure 2) [26]. This fluorescence data can be used to calculate the binding constant of the PP to the protein and the thermodynamic parameters, changes in enthalpy (ΔH) entropy (ΔS) and Gibbs free energy (ΔG), of the interaction. 

As an example, in milk, the PP curcumin predominantly binds to the CMs. Milk, containing both CMs and whey proteins, had a binding constant (K_b_) of 0.11 μM ^−1^, whereas pure CMs have a K_b_ of 0.22 μM^−1^, at a temperature of 25 °C [21]. Whey proteins, the other main proteins in milk, form aggregates upon heating, which attach to the surface of the CMs and increase the degree of curcumin binding to the CMs [21]. There is a great variety of milk proteins and milk protein ingredients with potential to bind PP, with different possible encapsulation efficiencies (EE).

Milk proteins are food-grade and widely researched as delivery systems for bioactive components. They can form complexes with hydrophobic and hydrophilic regions, which enables them to bind molecules, such as PP, with different polarities. Nutrition research often focuses on whey proteins, due to its high leucine content, which is positively linked to muscle recovery [28]. However, casein-based systems can be preferred from a techno-functional point-of-view, due to properties including high heat stability and surface activity that can be important in practical applications [29,30]. During digestion, the release of caseins from the stomach to the intestine is slower than whey proteins, due to coagulation of casein in the stomach, which results in a delay in digestion [31,32], potentially influencing the bioaccessibility and bioavailability of PP. The size of milk protein-PP complexes can have a wide range, with a minimum of 9 nm for BSA with quercetin and maximum of 100 μm for whey protein isolate aggregates with fruit juice [33,34,35,36]. The colloidal stability of the systems will depend on the size of the protein-PP complexes and systems with smaller particles will have a greater colloidal stability. It is also known that physicochemical properties of proteins, such as size and morphology can influence digestion [37], which could influence the protective effect of the protein on the PP. The main objective of this review was to provide a comprehensive overview of how the structure of different milk protein systems—which can depend on factors including processing history—may influence the binding of PP and the properties of resultant complexes. For example, PP binding may affect structural properties of protein-PP structures, as well as the antioxidant activity and digestibility characteristics of the PP. Such knowledge can play an important role in the application of polyphenols in milk-protein-containing systems.

## 2. Casein-Based Systems

### 2.1. Native Casein Micelles

**Figure 3:**
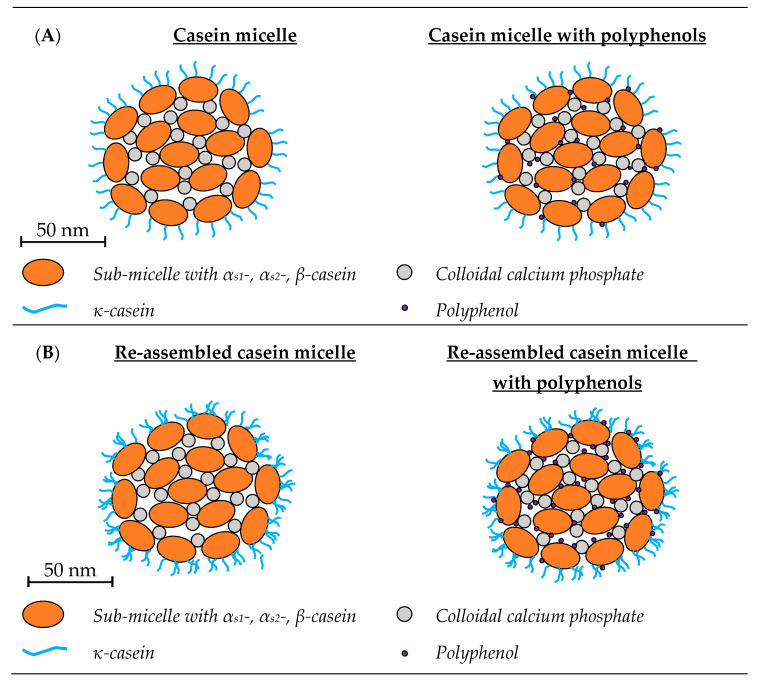

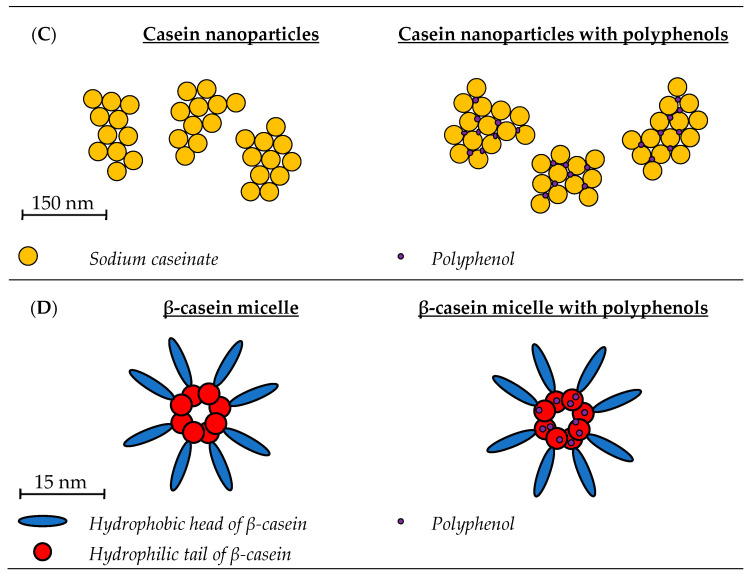
Schematic representation of casein systems with and without polyphenols. (**A**) Casein micelles are colloidal particles, consisting of casein sub-micelles, which are stabilised by colloidal calcium phosphate, resulting in formation of micellar complexes, which are surrounded by a hairy layer of κ-casein [20,57]. The interaction between polyphenols, such as curcumin and green tea flavonoids, and casein micelles are mainly hydrophobic [39,40,45]. More hydrophilic polyphenols, e.g., resveratrol, also interact with casein micelles through hydrogen bonds [38]. The interactions had no influence on the size and morphology of the casein micelles [39,41]. Hydrophobic polyphenols mainly interact with available hydrophobic patches within the core of casein micelles and more hydrophilic polyphenols with the hydrophilic areas in the casein micelles [38]. (**B**) Re-assembled casein micelles are artificially made micelles. There are made by adding tri-potassium citrate, calcium chloride and di-potassium hydrogen phosphate to sodium caseinate [58]. Their size and morphology are similar to native casein micelles, only their surface appears to be rougher, probably due to the presence of κ-casein aggregates [12,48]. The interaction between re-assembled casein micelles and polyphenols are similar to native casein micelles. For hydrophobic polyphenols, such as curcumin, the main interaction is hydrophobic and for more hydrophilic polyphenols, such as sesamol, the main interaction are hydrogen bonds [12,49]. (**C**) Casein nanoparticles are small aggregated protein structures made of sodium caseinate [48]. The interaction between sodium caseinate and the polyphenol resveratrol was through hydrogen bonds and hydrophobic interactions [59]. (**D**) β-casein has a strong amphiphilic character and will self-associate into micelles with a hydrophobic core and hydrophilic outer layer [60]. The polyphenols curcumin, naringenin and resveratrol mainly interact with β-casein micelles through hydrophobic interactions, in the hydrophobic core of the micelle [11,26,55].

The hydrophobic and hydrophilic regions of CMs can bind both hydrophobic and hydrophilic PP, such as curcumin and resveratrol (Figure 3A) [38]. The most widely studied PP in CM-based systems have been curcumin, resveratrol and green tea varieties (Table 1), with the structure of these PP provided in Figure 1. 

**Table 1:** Summary of the different casein-based encapsulation systems for polyphenols, including details of their particle size and encapsulation efficiency (EE), antioxidant activity assay and antitumor recorded cells. In which ABTS stands for azino-bis(3-ethylbenzothiazoline-6-sulfonic acid) and FRAP stands for ferric reducing ability of plasma. The abbreviations n.r. stands for not recorded and n.a. for not applicable.

Protein	Polyphenol	Particle Diameter (nm)	EE (%)	Antioxidant Activity Assay	Antitumor Recorded Cells	Ref.
Casein micelles	curcumin	138–150	n.r.	n.r.	n.a.	[38]
		<200	n.r.	n.r.	cervical	[39]
		n.r.	n.r.	n.r.	n.a.	[40]
		176–187	n.r.	n.r.	n.a.	[41]
		n.r.	n.r.	n.r.	n.a.	[42]
		n.r.	97	FRAP and ABTS	n.a.	[43]
	epigallocatechin gallate	n.r.	18–95	n.r.	n.a.	[44]
	green tea flavonoids	n.r.	n.r.	n.r.	n.a.	[45]
	resveratrol	138–150	n.r.	n.r.	n.a.	[38]
	tea catechins	n.r.	n.r.	n.r.	adenocarcinoma	[46]
	tea polyphenols	n.r.	n.r.	n.r.	n.a.	[47]
Re-assembled casein micelles	curcumin and quercetin	73–187	93–97	n.r.	breast	[12]
	epigallocatechin gallate	68	85	n.r.	n.a.	[48]
	sesamol	158–166	28–35	n.r.	n.a.	[49]
	quercetin	182–334	26–97	n.r.	n.a.	[50]
Casein nano particles	curcumin	169	83	ABTS	n.a.	[10]
		104–213	70–100	n.r.	colon	[51]
	curcumin and quercetin	187	>99	n.r.	breast	[12]
	epigallocatechin gallate	162–246	n.r.	n.r.	n.a.	[48]
with (2-hydroxypropyl-β-cyclodextrin)	quercetin	171–251	75–83	n.r.	n.a.	[52]
β-casein micelles	curcumin	n.r.	n.r.	ABTS	n.a.	[11]
	naringenin	~30	n.r.	n.r.	n.a.	[26]
		~20	n.r.	n.r.	n.a.	[53]
		9–12 and 79–359	n.r.	n.r.	n.a.	[54]
	resveratrol	n.r.	n.r.	n.r.	n.a.	[55]
		6–13	59 or 69	n.r.	n.a.	[56]

#### 2.1.1. Physicochemical Properties of Casein Micelle-Polyphenol Complexes

Sahu et al. studied the interaction between CMs and curcumin using fluorescence spectrophotometry. When comparing free curcumin in water to curcumin interacting with CMs, the low intensity broad fluorescence peak shifted from 540 to 500 nm, after excitation at 420 nm. This indicated that the main mechanism of interaction between curcumin and CMs were hydrophobic [39]. This was confirmed by Nadi et al., who performed a thermodynamic analysis of the binding process [40], the positive values for both ΔH, 35.00 kJ·mol^−1^, and ΔS, 0.23 kJ·mol^−1^, showed that the main type of interaction was hydrophobic. With the aid of scanning electron microscopy, atomic force microscopy, dynamic light scattering and small angle X-ray scattering, it was determined that binding of curcumin did not alter the structure of CMs [39,41]. Other PP also interact with CMs through hydrophobic interactions. The anilinonapthalene-8-sulfonate (ANS) binding study of Yuksel et al. investigated the binding interactions between CMs and green tea flavonoids [45]. The signal at 480 nm, after excitation at 390 nm, of samples with CM concentrations between 1 and 4% (*w*/*w*) and an ANS concentration of 135 mM was between 1073 ± 60 nm and 1295 ± 95 nm. Addition of 200 ppm phenols from green tea flavonoids decreased the signal for all CM systems to values between 393 ± 64 nm and 676 ± 18 nm. Green tea flavonoids decreased the available hydrophobic patches of CMs, demonstrating the role of hydrophobic interactions [45].

In the study of Yazdi and Corredig, the fluorescence intensity between 300 and 440 nm of diluted skimmed milk in permeate (1:20) was almost completely quenched when the curcumin concentration was increased from 2.5 and 45 μM, after excitation at 280 nm. This demonstrated that curcumin penetrates the CMs and can interact with proteins in the inner core of the CMs [21]. Yazdi et al. dissociated β-casein from CMs, creating CMs with a more hydrophobic core [38]. They measured the fluorescence emission spectra of curcumin between 450 and 650 nm after excitation at 330 nm, and for resveratrol between 350 and 550 nm after excitation at 320 nm. For curcumin (hydrophobic) an increase in β-casein removal from the CM resulted in an increase of the binding constant. Therefore, the authors state that the increase in available hydrophobic patches within the core of the CMs, caused by the removal of β-casein, resulted in the higher binding affinity for β-casein-depleted CMs compared to normal CMs. This suggests that depletion of β-casein from CMs results in a loosing of the CM structure, due to loss of phosphoserine clusters, leaving the CM association solely due to CCP. The loosening in structure could cause an increase of water molecules in the core of the micelles, which could lead to a more favourable environment for the formation of hydrogen bonds between the CM and resveratrol. The authors imply this is the reason that resveratrol has a higher binding affinity for β-casein-depleted CMs than CMs. This study demonstrated that the environment in the core of CMs has a strong influence on PP binding.

Both Sahu et al. and Khanji et al. found that the interaction between PP had no influence on the size (150–180 nm) and morphology of the CM [39,41]. To understand the thermodynamics of CM-curcumin binding, Hudson et al. performed partitioning, surface plasmon resonance and thermal degradation kinetic experiments [42]. It was shown that at 25 °C and pH 6.6, one CM was able to bind 18,000 curcumin molecules, indicating that the CM:curcumin ratio is the main factor influencing the EE. The studies reviewed in this article have different terms to describe the percentage of the total PP in the system bound to the proteins, e.g., EE [12], loading efficiency [24], total PP yield of particles [33], PP retention [43], and in this review the term EE will be used. An increase in epigallocatechin gallate (EGCG) concentration from 0.01 mg/mg protein to 0.50 mg/mg protein led to an EE decrease from 95 to 18%, which is probably due to saturation of the CM. Haratifar and Corredig confirmed this for EGCG, because the EE of EGCG in relation to CM depended on the EGCG concentration [44].

The effect of green tea PP on the stability of CMs was studied by van de Langerijt et al., by measuring the turbidity, sedimentation profile and surface hydrophobicity of CM dispersions with and without tea PP [47]. The tea PP reduced the turbidity loss of the CM dispersions over time, increased the integral transmission of the sedimentation profiles and decreased the available hydrophobic surface of the CMs. These results indicate that hydrophobic bonds between the tea PP and hydrophobic patches of the CM stabilise the CM structure.

#### 2.1.2. Effect of Casein Micelles on the Antitumor Activity of Polyphenols

Sahu et al. studied the cytotoxicity of CM-curcumin complexes [39]. This was achieved by measuring the amount of viable human cervical cancer cells (HeLa cells) after 48 h of exposure to free curcumin and CM-curcumin complexes, which resulted in an IC_50_ value of 14.85 and 12.69 µM, respectively. This indicates that CM-curcumin complexes have a higher cytotoxicity than free curcumin, which is probably due to higher curcumin absorbance of CM-curcumin complexes by the HeLa cells compared to free curcumin. Sahu et al. did not study the influence of digestion on the CM-curcumin complexes, which could potentially influence the cytotoxicity of both free and CM-curcumin complexes. In the study of Guri et al. two digestion models were used, a batch and dynamic digestion model, to study the influence of digestion on the cytotoxicity of EGCG in CM-EGCG complexes [46]. After digestion, the viability of adenocarcinoma cells was tested, and it was shown that the cytotoxicity after digestion with both models was preserved by the CMs. 

#### 2.1.3. Influence of Spray Drying and Rehydration on the Structure and Antioxidant Activity of CM-Curcumin Complexes

In the food industry, milk proteins are mostly processed to a powder form, due to storage and transport benefits, after which they are rehydrated to be used in their final formulation. The most common technique for drying is spray drying [61]. Khanji et al. produced a powder containing CM-curcumin complexes and investigated the influence of spray drying and rehydration on the structure and antioxidant activity of CM-curcumin complexes [43]. Small angle X-ray scattering measurements demonstrated that addition of curcumin did not alter the internal structure of the CMs, both before and after spray drying. The rehydration time of the powders was determined by measuring the time it takes to decrease the mean diameter (D50) to 0.7 nm, which corresponds to the D50 of native CMs, and was slightly shorter (i.e., faster rehydration) for the CM-curcumin complex powders (430 min) compared to the pure CM powders (470 min). This was likely because the main type of interaction between curcumin and CMs is hydrophobic and therefore less hydrophobic sites are available within the CMs, making the overall system more hydrophilic. The ferric reducing ability of plasma (FRAP) assay and 2,2′-azino-bis(3-ethylbenzothiazoline-6-sulfonic acid) (ABTS) assay indicated that the antioxidant activity of curcumin within the CM-curcumin complexes was not affected by the spray drying. These assays also indicated that storage of the spray dried complexes for 60 d at 40 °C preserved the antioxidant activity of the complexes when rehydrated. 

### 2.2. Sodium Caseinate

Sodium caseinate (SCN) is produced by first lowering the pH of skimmed milk to 4.6, which will lead to the dissociation of CCP from the CMs and result in isoelectric precipitation of casein, after which the whey is typically separated using a decanter centrifuge. A washing step removes residual lactose and minerals etc, and the caseins are resolubilised by adding sodium hydroxide and increasing the pH to 7 [62,63]. At neutral pH, SCN forms small aggregates with a diameter of approximately 20 nm, with the exact size depending on the ionic strength [64]. SCN can be used to create different structures, e.g., re-assembled casein micelles or casein nanoparticles. 

#### 2.2.1. Re-Assembled Casein Micelles

Re-assembled casein micelles (r-CMs) are made by addition of tri-potassium citrate, calcium chloride and di-potassium hydrogen phosphate to SCN. The salts create artificial CCP which enables SCN to form aggregates due to calcium-bridged phosphoserine-to-phosphoserine linkages, with a similar size (between 150 and 180 nm) and morphology to CMs [17,58,65,66,67]. These r-CMs are capable of binding hydrophobic components, thereby increasing their solubility in aqueous solutions and minimising their chemical degradation. If desired, hydrophobic components can be added to the SCN before it is converted to r-CMs. Therefore, hydrophobic components do not need to diffuse into the r-CMs structure but are incorporated upon r-CMs formation. This will potentially lead to incorporation of more hydrophobic compounds in r-CMs compared to native CMs. Semo et al., was the first to demonstrate that r-CMs bind 5.5 times more vitamin D_2_ (a hydrophobic vitamin) compared to native CMs, showing the potential of r-CMs as encapsulating agents [58]. Based on their findings, several studies were published on the encapsulation of PP by r-CMs, as shown in Table 1. A schematic representation of r-CMs with and without PP can be found in Figure 3B.

##### Physicochemical Properties of Re-Assembled Casein Micelle-Polyphenol Complexes

Within r-CMs there are both hydrophilic and hydrophobic regions, enabling r-CMs to interact with PP through hydrophilic and hydrophobic interactions [68]. Fluorescence spectroscopy studies were performed on r-CMs loaded with curcumin and sesamol [12,49]. The maximum emission wavelength of curcumin, after excitation at 287 nm, was at 348 nm independent of the curcumin concentration, when binding to r-CMs [12]. In contrast, the maximum emission wavelength of sesamol increased from 338 to 355 nm, after excitation at 280 nm [49]. This indicated that the main type of interaction between curcumin and quercetin with r-CMs was hydrophobic, while the main interaction with sesamol (a more polar PP) was mainly via hydrogen bonds. Thus, the polarity of PP primarily determines the type of interaction between r-CMs and PP. These findings indicate that r-CMs can potentially be used to bind both hydrophobic and hydrophilic PP. 

The morphology of the r-CMs was investigated by scanning electron microscopy and transmission electron microscopy [12,48,49]. When native CMs are visualised using electron microscopy a spherical shape with a smooth surface was observed [69]. The electron micrographs of r-CMs showed a spherical shape similar to native CMs, but their surface seemed to be rougher. This might be due to the κ-casein aggregates attaching to the surface of the r-CMs [12,48]. 

The size of r-CMs is influenced by several factors, such as the salt concentration, protein and PP concentration, type of PP, degree of heating and presence of surfactants [12,48,49,50]. In the study of Ghatak and Iyyaswami, r-CMs made with 0.5% (*v*/*w*) SCN had a mean diameter of 182 nm, an increase in SCN resulted in an increase of the mean diameter, with the largest diameter of 296 nm at 5% SCN [50]. It is suggested that small protein aggregates attach to the r-CMs, thereby increasing the particle size.

The EE of sesamol by r-CMs was researched by Santos Basurto et al., who observed that an increase in SCN concentration led to an increase in EE [49]. The highest EE of 36% was found at a SCN concentration of 5% (*w*/*v*) and a sesamol concentration of 1 mg/mL. This increase in EE was probably due to an increase of available hydrophobic patches (caused by the increase in SCN concentration), which can interact with sesamol. Ghatak and Iyyaswami observed a different relationship between the EE of quercetin and SCN concentration [50]. An increase of SCN concentration led to a decrease in EE and the maximum EE of ~84% was found with 0.5% (*w*/*v*) SCN. The authors attributed this to the differences in size and structure of r-CM caused by the different SCN concentrations. The size of the r-CMs was smaller with lower SCN concentrations; indeed, these data suggest that the smaller r-CMs formed at lower SCN concentrations have a higher EE. It is suggested that at higher protein concentrations the proteins favour hydrophobic interactions with each other instead of with quercetin, leading to the increase in particle size and decrease in EE of r-CMs. The difference in influence of SCN concentration on the EE of curcumin and quercetin was probably due to the difference in the conditions under which the r-CMs were formed. This may have led to differences in the structure of the r-CMs, which influenced the EE. It might also be that the two different relationships between the SCN concentration of r-CMs and the EE of sesamol and quercetin was due to the different properties of the PP. The type of PP incorporated in the r-CMs also influenced the EE of the system, similar r-CMs incorporating curcumin, EGCG and quercetin, had an EE of 97, 85 and 93%, respectively [12,48]. Even though the EE of all these PP was high, the results indicate that the EE is dependent on the type of PP, which could be due to the difference in polarity and binding coefficient of the PP.

The influence of quercetin concentration (12–22 μM) on the EE of r-CMs made with 0.5% (*w*/*v*) SCN was studied by Ghatak and Yyaswami, with a decrease in EE from 97 to 7% observed when quercetin concentration increased from 12 to 22 μM [50]. The authors suggest that this decrease in EE was due to limitations in the amount of quercetin that could interact with the hydrophobic patches of the r-CMs.

The influence of pH on the EE of curcumin, EGCG and quercetin by r-CMs has also been studied [12,48,50]. With pH values greater than 7, the EE of EGCG and quercetin decreased, whereas the EE of curcumin was largely unchanged. An increase in pH increases the overall negative charge of r-CMs, which results in a looser micellar structure, due to electrostatic repulsion [48], causing EGCG and quercetin dissociation and formation of anionic species, with consequent reductions in EE. However, the EE of curcumin was not influenced by the increase of pH, suggesting that influence of pH on the EE of r-CMs is dependent on the type of PP. 

When the effect of storage time of the EE of r-CMs loaded with curcumin, EGCG and quercetin was studied, a period of 21 d had almost no influence on the EE of r-CNs. This indicates that the type of PP does not influence the effect of time on the EE [12,48].

Ghatak and Iyyaswami studied the influence of tri-potassium citrate, calcium chloride and di-potassium hydrogen phosphate on the EE of quercetin in r-CMs [50]. Tri-potassium citrate is a calcium chelating agent and actively binds calcium. Concentrations up to 1 M increase the EE from 55 to 83%, with higher concentrations decreasing the EE. Low concentrations of citrates are known to increase the number of cross-links within r-CMs, whereas higher concentrations cause destabilisation and removal of CCP from the r-CMs [70]. The increase in cross-linking enabled the growth of the colloidal mass of the r-CMs, which may have resulted in more extensive quercetin binding and increased the EE. In contrast, the destabilisation of r-CMs resulted in less quercetin binding and a decrease in EE. The influence of calcium chloride concentration between 0 and 1 M was studied and the highest EE of 87% was measured at 0.5 M. Higher concentrations than 0.5 M led to aggregation of r-CMs, resulting in precipitation of the r-CMs, with associated reductions in EE. Di-potassium hydrogen phosphate was used to stabilise the r-CM structure, but concentrations between 0 and 1 mM had no influence on the EE of r-CMs. 

##### Influence of Re-Assembled Casein Micelles on the Antitumor Activity of Polyphenols

The PP curcumin and quercetin are known cytotoxic molecules. The work of Ghayour et al. studied the effect of PP binding on r-CMs, the impact of digestion on the PP in r-CM-PP complexes, and the effect of r-CMs on the cytotoxicity of curcumin and quercetin, by measuring the cell viability of MCF-7 human breast cancer cells [12]. It was shown that binding of curcumin and quercetin by r-CMs improved their cytotoxicity. Digestion of these loaded r-CMs improved the cytotoxicity even further, leading to the conclusion that r-CMs improve the delivery of curcumin and quercetin and can potentially be used as part of novel drug delivery systems. 

When comparing the different studies on PP binding by r-CMs it becomes clear that once the optimal parameters are identified (e.g., SCN concentration, pH, salt concentration), the EE of r-CMs can be high for both hydrophobic and hydrophilic components. This, together with the observation that r-CMs improve the cytotoxicity of curcumin and quercetin, implies strong potential for r-CMs as PP delivery systems. 

#### 2.2.2. Casein Nanoparticles

Casein nano particles (CNP) are small protein structures made of SCN with an average diameter between 100 and 250 nm. In recent literature, four different methods were used to create casein nanoparticles to study their binding of PP; a representation of their general structure can be found in Figure 3C. In the simplest method, SCN was dispersed simultaneously with quercetin, curcumin or EGCG in deionised water at 25 °C [12,48]. In the method of Pan et al. SCN was dispersed in 40% (*v*/*v*) aqueous ethanol and heated for 5 min at 60 °C, after which curcumin was added [10]. The unbound curcumin was removed by centrifugation and the CNP were spray dried. The study of Pan et al. involved preparation of pH-driven CNP by dispersing SCN in water at room temperature, adjusting the pH to 12 with NaOH, adding curcumin and adjusting pH to 7 with HCl [51]. Peñalva et al. added 2-hydroxypropyl-β-cyclodextrin (2-h-β-c) to CNP, to prevent the metabolism of quercetin prior to absorption in the intestine [52]. The CNP were produced by dispersing SCN, lysine and 2-h-β-c in water, after which quercetin (dissolved in ethanol) and calcium chloride were added.

##### Physicochemical Properties of Casein Nanoparticle-Polyphenol Complexes

The binding of resveratrol by SCN was studied by Acharya et al. by measuring fluorescence quenching [59]. The excitation wavelength was 280 nm, after which the emission intensity between 300 and 600 nm was measured. SCN had an emission band around 350 nm, which was quenched (decrease in intensity), with an increasing resveratrol concentration. Further thermodynamic analysis of the results gives a negative value for the ΔH, −21.2 kJ·mol^−1^, a positive value for the ΔS°, 37.9 kJ·mol^−1^. The negative value for ΔH, indicates the presence of hydrogen bonds and positive value for ΔS indicates the presence of hydrophobic interactions [59].

The diameter of CNP is typically in the range 100–250 nm, with the exact size dependent on the preparation method. Dissolving 1% (*w*/*w*) SCN in water resulted in CNP with an average diameter of 162 nm [48] or 187 nm [12], a heat treatment of 20 s at 74 °C increasing the average diameter to 246 nm [48]. The spray dried CNP, made in aqueous ethanol, had an average diameter of 169 nm, which was similar to the size of the unheated CNP [10]. When the CNP were prepared by the method of Pan et al., the average diameter of the CNP depended on the initial curcumin concentration in the system; for example, a decrease in diameter from 213 to 104 nm, was observed when the curcumin concentration increased from 0 to 2 mg/mL [51]. The CNP in the study of Peñalva et al. had a diameter of 138 nm without quercetin, 251 nm with quercetin and 171 nm with quercetin and 2-h-β-c [52]. 

All CNP had a high EE between 70 and effectively 100%. The EE of quercetin and curcumin by CNP, made with 0.5% (*w*/*w*) SCN and a 1:1 molar ratio of quercetin or curcumin with SCN, was determined in the study of Ghayour et al., and was higher than 99%. This was attributed to the high number of hydrophobic interactions and hydrogen bonds between the PP and SCN [12]. The ethanol spray dried CNP made with 2% (*w*/*w*) SCN and 0.5% (*w*/*w*) PP, had an EE of 83%, and significantly higher solubility of curcumin in water, 11 ng/mL in water compared to 137 μg/mL in a colloidal dispersion with 1.25% (*w*/*v*) CNP [10]. Just like the size, the EE of the pH-driven CNP prepared in the study of Pan et al. depended on the curcumin concentration [51]. The CNP prepared with 2% (*w*/*w*) SCN decreased the EE from 100 to 70%, when the curcumin concentration increased from 0.4 to 2 mg/mL. However, the total amount of curcumin bound by the CNP increased with increasing curcumin concentration. The CNP in the study of Peñalva et al. increased in EE from 75 to 83% when 2-h-β-c was added [52]. 

##### Functional Properties of Casein-Polyphenol Nanoparticles

Using the ABTS assay, Pan et al. reported that binding of curcumin increased the antioxidant activity of aqueous solutions from <1.0 to 8.9 mM [10]. Free curcumin precipitates in water, resulting in the low antioxidant activity of the sample. Binding to CNP helped to keep the curcumin solubilised, which increased the antioxidant activity of the sample. 

In the study of Malekhosseini et al., a shelf-life stress test was performed on the CNP to measure the effect of CNP on EGCG degradation [48]. This was done by heating the CNP to 45 °C for 31 d and extracting EGCG from the CNP with a diluted ethanol solution at different time points. The amount of extracted EGCG was determined by measuring the UV absorbance of the ethanol extract at 274 nm. It was shown that CNP did not influence the degradation rate of EGCG, possibly attributable to the open structure of the CNP. Ghayour et al. performed the same experiment on curcumin and quercetin and measured the absorbance of the ethanol extract at 468 and 373 nm, respectively [12]. Curcumin was better protected from degradation over a time span of 28 d compared to quercetin, the authors suggested that this was due to the thermal sensitivity of quercetin at 45 °C.

##### Influence of Casein Nanoparticles on the Bioavailability of Polyphenols

Peñalva et al. studied the in vitro digestive release of quercetin, and the influence of CNP (both with and without 2-h-β-c) on this release [52]. The samples were subjected to 2 h of gastric digestion at pH 1.2 and 24 h intestinal digestion at pH 6.8. The CNP, both with and without 2-h-β-c, released 20% of the EGCG during the gastric digestion. After the first 4 h of intestinal digestion, the release rate of the CNP with and without 2-h-β-c was 60 and 80%, respectively. The authors suggested that this decrease in release rate caused by 2-h-β-c was due to formation of CNP with a more compact matrix. Peñalva et al. also performed an in vivo digestion study, in which the quercetin concentration present in blood plasma of rats was measured, after being fed samples with 2.5 mg/mL quercetin, in the form of free quercetin, CNP or CNP with 2-h-β-c [52]. Consumption of free quercetin resulted in a low concentration of quercetin in the blood plasma, which rapidly decreased, resulting in a bioavailability of 4%. The concentration of quercetin in the blood plasma after consumption of quercetin bound to CNP and CNP with 2-h-β-c was significantly higher compared to consumption of free quercetin. After consumption of quercetin bound to CNP, the concentration of quercetin in the blood plasma decreased slowly for 6 h after consumption. After 24 h, only a low amount was detected, with an associated bioavailability of 12%. The quercetin concentration in the plasma after consumption of CNP with 2-h-β-c remained high up until the last measurement 72 h after consumption, resulting in a bioavailability of 37%. This improvement in bioavailability might have been due to partial protection from gastric digestion, which resulted in more quercetin absorption in the intestine. 

##### Effect of Casein Nanoparticles on the Antitumor Activity of Polyphenols

The in vitro cell proliferation assay, with colon cancer cells (HCT-116) of Pan et al. determined the effect of binding curcumin (1, 5, 10 and 20 μg/mL) by CNP on the cell viability [51]. As expected, an increase in curcumin resulted in a decrease in cell viability. The bound curcumin also had a lower cell viability (~50%) than the free curcumin (~75%) at the highest curcumin concentration of 20 μg/mL; this was likely due to the improved solubility of curcumin by its binding to casein nanoparticles. Ghayour et al. measured the cell viability of breast cancer cells (MCF-7) when exposed to curcumin or quercetin bound to CNP [12]. As in the previous study, an increase in PP resulted in decreased cell viability. Binding of curcumin at a concentration of 1800 μM to CNP resulted in a greater decrease of cell viability (~15%) than binding of quercetin to CNP (50%) at a concentration of 1800 μM.

### 2.3. β-Casein Micelles

The protein β-casein has 209 amino acids, the unique combination of which results in a molecular weight of ~24 kDa and an isoelectric point of 4.9. Its highly negatively charged hydrophilic N-terminus and weakly positive charged hydrophobic C-terminus, give β-casein an amphiphilic character [16,71,72]. The hydrophobic interactions between the C-terminuses of the β-casein proteins leads to self-association, resulting in formation of small micelles, called β-casein micelles (β-CMs). Their average diameter is typically in the range 20–30 nm and the β-CMs have a hydrophobic core and hydrophilic outer layer [60]. Due to their hydrophobic core, it is suggested that they present strong potential for nano-encapsulation of hydrophobic molecules, such as PP (Figure 3D) [11,73]. 

#### 2.3.1. Physicochemical Properties of β-Casein Micelle-PP Complexes

Moeiniafshari et al. performed fluorescence spectroscopy studies on the interactions between β-CMs and naringenin at neutral pH, with the results used to perform a thermodynamic analysis on the binding interactions [26]. It was shown that the binding between β-CM and naringenin is mainly caused by hydrophobic interactions, but also by van der Waals interactions and hydrogen bonds. Other fluorescence binding studies confirmed that the interaction between β-CM with curcumin and resveratrol was also predominantly hydrophobic [11,55].

The size and morphology of the β-CM-PP complexes depended on the pH of the system. Li et al. showed that at pH 7, the amino acids tryptophan and tyrosine moved from an apolar to a more polar environment, when binding with naringenin, indicative of a change in conformation of the β-CMs [53]. In contrast, at pH 2, this change in tryptophan and tyrosine to a more polar environment did not occur. The C-terminus loses most of is negative charge at pH 2, which reduces its hydrophobic nature, leading to the formation of larger β-CM-naringenin complexes [53]. At pH 7, the addition of NaCl should screen the charges present on β-CMs, leading to formation of larger β-CM-naringenin complexes, but the main peak in the size distribution remained at 28 nm. It is suggested this is caused by prevention of micelle formation, due to the screening of the negative charges of the N-terminus. 

At neutral pH, temperature influences the size of the β-CMs without PP. At temperatures less than 15 °C, they have a relatively small diameter, compared to at temperatures greater than 15 °C. This is due to the reduced strength of hydrophobic interactions at lower temperatures, with most β-casein being present in the monomeric form. At higher temperatures the strength of the hydrophobic interactions increases, and the monomers will turn into micelles [60,74]. When naringenin was added to β-CMs the size of the micelles remained constant between a temperature of 5 and 55 °C [53]. Li et al. attempted to stabilise the β-CM-naringenin complexes with genipin [54]. The genipin cross-linked the β-caseins within the micelles by intra-micellar covalent interactions. The cross-links improved the colloidal stability of the β-CM-naringenin complexes at pH 2.0. The genipin cross-links also decreased the release rate of naringenin from β-CM. 

Esmaili et al. found that β-CMs improve the solubility of curcumin in aqueous solutions up to 2500 times [11]. The EE of resveratrol depended on the β-casein concentration and type of resveratrol isomer. An increase in β-casein resulted in an increased EE, with the highest value reported at 200 µM β-casein, and 69% for trans-resveratrol and 59% for cis-resveratrol [56].

#### 2.3.2. Functional Properties of β-Casein Micelle-PP Complexes

Using a ABTS assay, Esmaili et al. demonstrated that the antioxidant activity of β-CM-curcumin complexes with 20 mM curcumin had a higher antioxidant activity than 120 mM curcumin dissolved in 10% ethanol; this indicated that binding of curcumin by β-CMs significantly improved the antioxidant activity of curcumin [11]. An in vitro cytotoxicity study showed that β-CM-curcumin complexes have a higher cytotoxicity than free curcumin for human leukaemia cells (cell line K-562); the IC_50_ value for β-CM-curcumin complexes and free curcumin was 17.7 and 26.5 mM, respectively [11].

## 3. Whey Protein-Based Systems

**Figure 4:**
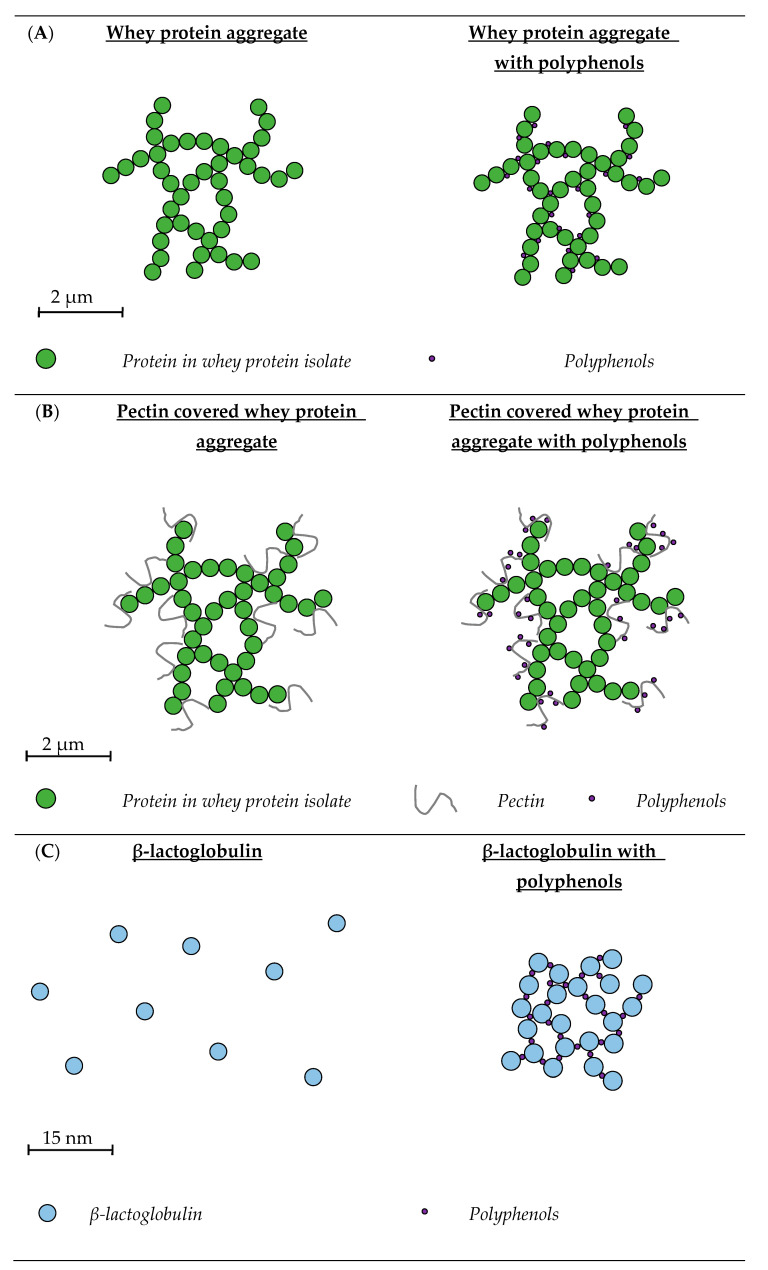

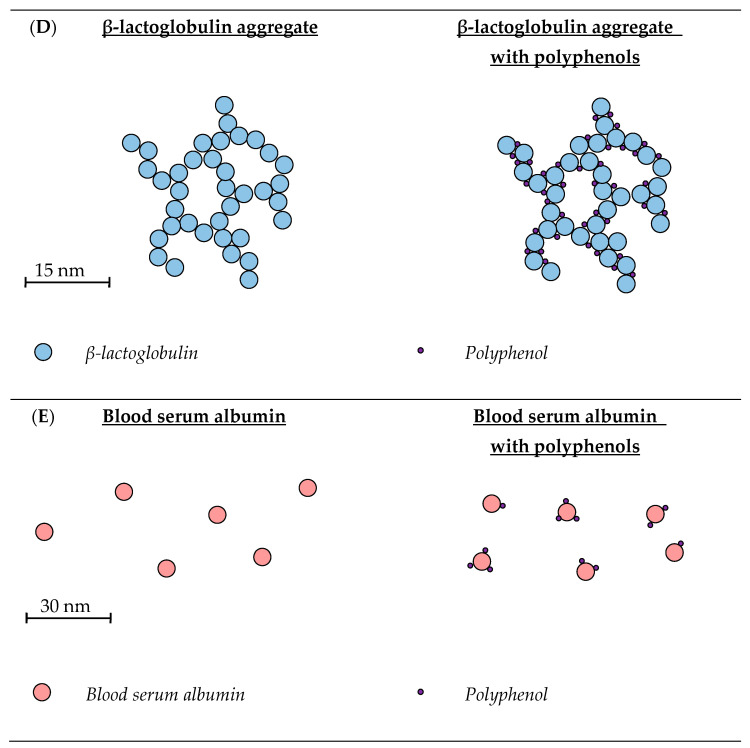
Schematic representation of whey protein systems with and without polyphenols. (**A**) Whey protein aggregates are made by lowering the pH of the system to 4.5, close to the isoelectric point of the whey proteins, which will lead to the formation of protein aggregates, due to due to minimal protein charge–charge repulsions [33,90]. Polyphenols interact with whey protein aggregates through hydrophobic interactions [33]. The size of the whey protein-PP aggregates depends on the pH, type of polyphenol and concentration of polyphenols added to the system [33,34]. (**B**) Pectin covered whey protein aggregates are made by heating whey protein isolate dispersions, which will lead to protein denaturation and aggregate formation. A solution with pectin was added to the whey protein aggregates and the pH was lowered to 3.2, giving the aggregates a positive and pectin a negative charge, resulting in the formation of pectin covered whey protein aggregates [78]. It is suggested that polyphenols, like anthocyanins, bind to the surface of pectin covered whey protein aggregates [78]. (**C**) β-lactoglobulin is a globular protein and around neutral pH it is mainly present as a non-covalent linked dimer [91]. Addition of catechins to β-lactoglobulin leads to complex formation, an increase in polyphenol concentration results in an increase in the size of these complexes [82]. The interactions between catechin and β-lactoglobulin are both hydrophobic and hydrophilic [83]. (**D**) Heating of β-lactoglobulin leads to denaturation, resulting in heat induced aggregates which are able to bind more epigallocatechin gallate then unheated β-lactoglobulin. This is probably the result of more available hydrophobic amino acids, due to heat induced denaturation, which have a hydrophobic interaction with epigallocatechin gallate [84]. (**E**) Blood serum albumin is a relatively large whey protein which is highly structured but flexible [75]. They can bind to polyphenols such as quercetin, mainly through hydrophobic interactions and tea catechins through both hydrophobic and hydrophilic interactions [35,89]. The size of blood serum albumin with and without quercetin is similar to each other [35,36].

Around 20% of the proteins in bovine milk consist of whey proteins, of which the main types are, β-lactoglobulin (β-Lg), α-lactalbumin (α-La), blood serum albumin (BSA) and immunoglobulins (Igs), which represent ~60, 20, 10 and 10%, respectively [75]. They are globular proteins and unfold when heated at temperatures greater than their denaturation temperature. Depending on the pH, denaturation leads to formation of different aggregate shapes: spherical particles, flexible strands and semi-flexible fibrils [76]. There are numerous studies investigating different whey proteins as delivery systems for PP (Table 2, Figure 4).

**Table 2:** Summary of the different whey protein-based encapsulation systems for polyphenols, including details of their particle size and encapsulation efficiency (EE), antioxidant activity assay and antitumor recorded cells. In which ABTS stands for azino-bis(3-ethylbenzothiazoline-6-sulfonic acid), DPPH stands for 2,2-diphenyl-1-picrylhydrazyl, FRAP stands for ferric reducing ability of plasma and ORAC stands for oxygen radical absorbance capacity. The abbreviations n.r. stand for not recorded and n.a. for not applicable.

Protein	Polyphenol	Particle Diameter (nm)	EE (%)	Antioxidant Activity Assay	Antitumor Recorded Cells	Ref.
Whey protein isolate	blackcurrant juice, cran-berry juice and musca-dine grape juice	5000, 30,000 and 100,000	28–84	n.r.	n.a.	[33]
	blueberry juice and cranberry juice	1000–100,000	21–46	n.r.	n.a.	[34]
with pectin	Anthocyanin-rich extract Grape seed extract, hibiscus extract, tannic acid and chatechin	175–204	35 or 55	n.r.	n.a.	[77]
		2000–17,000	n.r.	n.r.	n.a.	[78]
β-lactoglobulin (unheated)	(+) catechin	<458	n.r.	ABTS and ORAC	n.a.	[79]
	green tea extract	33	n.r.	n.r.	n.a.	[80]
		33–955	n.r.	n.r.	breast, colon, glioma, kidney, lung, melanoma, ovarian and prostate	[81]
		15–200	62–85	n.r.	n.a.	[82]
	tea catechins	n.r.	n.r.	n.r.	n.a.	[83]
β-lactoglobulin (heated)	epigallocatechin gallate	<50	n.r.	n.r.	n.a.	[84]
		<50	60–70	n.r.	n.a.	[85]
		43	40	FRAP and ORAC	n.a.	[86]
		2–50	74	n.r.	n.a.	[87]
		31	59	n.r.	adenocarcinoma, cervical, esophageal carcinoma, gastric, lung, melanoma ovarian and prostate	[88]
Blood serum albumin	quercetin	9–12	n.r.	ABTS and DPPH	n.a.	[35]
	quercetin	~10 nm	n.r.	ABTS and DPPH	n.a.	[36]
	tea catechins	n.r.	45–65	n.r.	n.a.	[89]

### 3.1. Whey Protein Isolate

Whey protein isolate (WPI) is a highly pure source of whey protein, with protein content ≥90% and is prepared using ion exchange chromatography technology to selectively enrich proteins from liquid whey [92], with the four main whey proteins in WPI present in the same ratio as in milk [14].

#### 3.1.1. Whey Protein Isolate Aggregates

Schneider et al. used WPI aggregates to bind PP (Figure 4A). WPI was added to blackcurrant, cranberry and muscadine grape juice with different PP concentrations, 50, 250 or 500 mg of PP/L juice, to obtain a solution with a final protein content of 20% (*w*/*w*) [33]. Diaz et al. produced the same aggregates, but added different WPI concentrations (10, 15 and 20%, *w*/*w*, protein) to blackberry and cranberry juice with 1800 PP mg/L juice [34]. The pH in both studies was adjusted to 4.5 and after 30 min the WPI aggregates binding PP were separated by centrifugation, after which they were redispersed in water [34]. These redispersed WPI-PP complexes were spray dried in the study of Schneider et al. and freeze-dried in the study of Diaz et al. [33,34]. In both studies, the size of the particles was measured before and after spray/freeze drying. The size of the WPI-PP aggregates depended on the pH, type of PP and the concentration of PP and protein, but in general, the size was in the range of 2–200 µm.

The EE of the WPI aggregates depended on the type of PP and the PP and protein concentration, for all types of PP, the EE decreased with increasing PP concentration. This is probably because PP were able to interact with the available hydrophobic areas of WPI aggregates. Once these hydrophobic areas were occupied it was less likely that PP would bind to the whey proteins. The maximum EE of 84% (*w*/*w*) was found for cranberry juice, with a PP concentration of 50 mg/L and protein concentration of 20% (*w*/*w*) WPI.

#### 3.1.2. Whey Protein Isolate-Pectin Aggregates 

Thongkaew et al. and Arroyo-Maya and McClements prepared PP delivery systems of WPI-pectin aggregates (Figure 4B) [77,78]. In the study of Thongkaew et al., a WPI and pectin dispersion was prepared by dissolving WPI (5% *w*/*w*) and apple pectin (2% *w*/*w*) in a 10 mM sodium phosphate buffer of pH 7 [78]. The WPI dispersion was heated for 40 min at 65 °C to form WPI aggregates. These WPI aggregates and the pectin dispersion were combined in a 1:1 ratio, after which the pH was adjusted to 3.2. This gave a positive charge to the WPI aggregates and a negative charge to the pectin, resulting in WPI-pectin aggregate formation due to electrostatic interactions. Different concentrations of grape seed extract, hibiscus extract, tannic acid and catechin (1, 3 and 5 mg/mL) were added to the WPI-pectin aggregates. The same WPI aggregates were also made without pectin for comparison. In the study of Arroyo-Maya and McClements, WPI and sugar beetroot pectin were dissolved separately in water and thereafter combined to create a dispersion with 0.5% (*w*/*w*) WPI and 0.25% (*w*/*w*) pectin [77]. The pH was adjusted to 5.8 and the solution heated at 90 °C for 5 min to form WPI aggregates. Afterwards, the pH was reduced to 4, creating WPI-pectin aggregates. An anthocyanin-rich extract (0.30 and 0.60 mg/mL PP) was added either before or after the heating step, to yield particles with different sizes. Thongkaew et al. reported particle sizes around 12.4 µm, depending on the PP type and concentration [78]. This size was similar to the size of the WPI aggregates made without pectin [33,34]. However, Arroyo-Maya and McClements reported a size between 175 and 204 nm [77]. These WPI-pectin aggregates showed an increase in size and EE when the PP were added before heating. The EE increased with decreasing PP concentrations as well, which was also found in the WPI aggregates without pectin [33,34]. The maximum EE of 55% was reported for WPI-pectin aggregates to which 0.3 mg/mL anthocyanins were added before heating. 

The influence of complex formation on the colour stability of anthocyanins, once ascorbic acid was added, was studied over a period of 7 d. It was shown that complex formation did not improve the colour stability of anthocyanins, which was probably because ascorbic acid was small enough in size to diffuse into the WPI-pectin aggregates. Both WPI aggregates and WPI-pectin aggregates were able to form protein-PP complexes. The PP present in one portion of blueberries (75 g) could be served in 12 g of protein-PP complexes, and the PP in one glass of cranberry juice (236 mL) in 17 g protein-PP complexes [34]. An advantage of WPI-pectin aggregates over WPI aggregates is the lower protein precipitation capacity, which resulted in a more stable system [78]. However, pectin WPI-pectin aggregates demonstrate greater sensitivity to pH than WPI aggregates, therefore, choosing one over the other depends on the desired system properties. 

### 3.2. β-Lactoglobulin

The main whey protein in milk is β-Lg, which contains 162 amino acids and has a molecular weight of approximately 18 kDa [91]. Its isoelectric point is ~4.6 or 5.2, depending on the genetic variant and its denaturation temperature is ~78 °C [22,93]. Around 50% of the total protein in WPI is β-Lg, and this section will focus on pure β-Lg systems.

#### 3.2.1. Unheated β-Lactoglobulin

Most research on unheated β-Lg-PP complexes (Figure 4C) has been focused on green tea PP, which consist mainly of four different types of catechin (+)-catechin (C), (−)-epicatechin (EC), (−)-epicatechin gallate (ECG) and EGCG. For the preparation of these β-Lg-PP complex systems, β-Lg and green tea powder extract were generally dissolved separately in a 0.01 M phosphate buffer of pH 6.0 at room temperature, stirred for 30 min, and mixed in the desired ratio [80,81,82]. 

##### Physicochemical Properties of Unheated β-Lactoglobulin

Kanakis et al., investigated the interactions between unheated β-Lg and the green tea catechins C, EC, ECG and EGCG, with the aid of different spectroscopy studies (Fourier transform infrared, circular dichroism and infrared) and molecular docking analysis [83]. It was shown that higher levels of OH groups on the PP led to greater binding to β-Lg, in the order EGCG > ECG > EC > C. The types of binding between β-Lg and tea PP are both hydrophobic and hydrophilic, with the binding of green tea catechins leading to the formation of more β-sheets and α-helixes in β-Lg.

Binding of PP by unheated β-Lg resulted in an increase in particle size, with addition of 0.1% (*w*/*w*) PP to 0.3% (*w*/*w*) β-Lg at pH 6.0, resulting in a particle diameter increase from 6 to 33 nm [79,80,81,82]. It was also found that pH, PP concentration and polysaccharide addition influenced the size of the complexes. At pH 3, the addition of 0.1% (*w*/*w*) PP to 0.3% (*w*/*w*) β-Lg increased the size from 3 to 142 nm and from 7 to 955 nm at pH 4.5. It is suggested that this large increase at pH 4.5, is associated with the isoelectric point of β-Lg. This point is at pH 4.5, in which β-Lg is mainly present in the form of oligomers which are more sensitive to association with PP [81]. The increase of PP concentration from 0.125 to 1% (*w*/*w*) increased the diameter of the β-Lg-PP complexes from 15 to 200 nm [82]. Addition of the polysaccharide pectin (0.38% *w*/*w*) to a system with 0.3% (*w*/*w*) β-Lg and 0.02% (*w*/*w*) PP at pH 4, resulted in a size increase from 5 to 458 nm. However, addition of the polysaccharide chitosan had no influence on the size distribution [79]. At pH 4, β-Lg, pectin and chitosan have a positive, negative and positive net charge, respectively. This causes attraction between β-Lg and pectin, resulting in complex formation and the particle size increase. There will be a repulsion between β-Lg and chitosan preventing complex formation and no influence on the particle size will be observed. The influence of PP concentration on the EE of 0.3% (*w*/*w*) β-Lg was studied by Rodríguez et al. [82]. An increase in PP concentration from 0.125 to 1.0% (*w*/*w*), decreased the EE from 85 to 62%. However, the quantity of PP bound on a protein basis increased with increasing PP concentration [79]. 

To use unheated β-Lg as a delivery system, the PP need to retain their antioxidant activity upon binding to β-Lg. This was studied by Oliveira et al., using a ABTS and oxygen radical absorbance capacity (ORAC) assay, who found that binding did not reduce the PP antioxidant activity [79]. They also found that pectin or chitosan had no influence on the antioxidant activity of the PP in unheated β-Lg-PP complexes. 

##### Effect of Unheated β-Lactoglobulin on the Antitumor Activity of EGCG

Von Staszewski et al. investigated the influence of β-Lg-TPP complexes on the antiproliferative activity of different tumour cell lines (breast, colon, glioma, kidney, lung, melanoma, ovarian and prostate cancer cells) [81]. The antiproliferative activity is expressed as the concentration of TPP needed to achieve total growth inhibition in the tumour cells. The concentration of TPP to achieve total growth inhibition in melanoma, kidney, and ovarian tumour cells was lower if the TPP were present in β-Lg-TPP complexes. For breast, colon, glioma, lung and prostate tumour cells the concentration was lower for free TPP. 

#### 3.2.2. Heated β-Lactoglobulin

Shpigelman et al. studied the properties of heat induced β-Lg-EGCG complexes (Figure 4D). Nanoaggregates were made by heating a solution of β-Lg to 75–85 °C for 20 min, after which a EGCG solution was added. Their size, EE, level of protection against EGCG oxidation and sensory properties were investigated [84]. Once these were determined the potential influence of the β-Lg-EGCG complexes on the digestive release, bioaccessibility and bioavailability of EGCG were studied [85,86]. The influence of β-Lg-EGCG complexes on bio-functional properties, antitumor activity and biological efficacy in a high fat diet (HFD) obesity mice model, have also been studied [87,88]. 

##### Physicochemical Properties of Heated β-Lactoglobulin

Shpigelman et al. were the first to prepare heat induced β-Lg-EGCG complexes. In this study, binding of EGCG to heated and unheated β-Lg were compared using spectrofluorometric and UV absorbance binding [84]. Unheated β-Lg had an K_b_ of 1.05 × 10^5^ M^−1^ and heated β-Lg one of 3.70 × 10^5^ M^−1^, demonstrating that heated β-Lg bound more EGCG. This was likely due to unfolding of the β-Lg upon heating, resulting in increased exposure of hydrophobic amino acids, with these hydrophobic amino acids able to bind a greater number of EGCG molecules. The unfolding of β-Lg and binding of EGCG resulted in a colloidally stable system of which the particles had a size < 50 nm [84,85].

A β-Lg concentration of 0.5% (*w*/*w*) and 1% (*w*/*w*) had an EE of ~58.5% and ~70.5%, respectively. This was independent of the two tested ECGG:β-Lg molecular ratios, 2:1 and 8:1 [85]. It is suggested that this increase in EE at higher β-Lg concentrations was due to the higher probability of EGCG and β-Lg interacting in more crowded systems. However, the EE of the heat induced β-Lg nanoaggregates prepared in the study of Zagury et al. was lower (40.1%), even though the nanoaggregates were made with a similar ECGC: β-Lg ratio [86]. The binding of EGCG reduced EGCG oxidation 33-fold for the first 48 h and 3.2-fold after 8 d [84]. 

##### Influence of Heated β-Lactoglobulin on Bioaccessibility and Bioavailability of EGCG

Digestion was studied by Shpigelman et al. and Zagury et al. [85,86]. Both articles studied in vitro gastric digestion by adding pepsin to the β-Lg nanoaggregates, lowering the pH to ~2.5 and analysing the intact β-Lg at different time points. An increase in digestion time reduced the β-Lg intensity on a polyacrylamide electrophoresis gel, from 100 to 68% after 2 h [85]. The study of Zagury et al. showed no degradation of β-Lg during the first 60 min of gastric digestion [86]. The difference in β-Lg digestion might be due to the different conditions used for digestion across the two studies. The study of Zagury et al. found no release of EGCG after 60 min of digestion [86]. When the release of EGCG during in vitro gastric digestion was studied by Shpigelman et al. it was shown that after 90 min, the β-Lg-EGCG complexes started to release EGCG, and after 2 h, 25% was released [85]. The reason that EGCG was not released in the first 90 min was due to binding of EGCG to the β-Lg peptides; interestingly, only when peptides were digested to a molecular weight less than 3 kDa, EGCG started to be released [85]. 

Zagury et al. also studied the intestinal digestion, which was performed by adding simulated intestinal fluid, bile salts, taurocholic acid and sodium glycodeoxycholate to the gastric digested sample and adjusting the pH to 6.5, where it was maintained for 120 min at 37 °C [86]. Rapid degradation of β-Lg occurred during the intestinal digestion, and compared to samples with free EGCG, the amount of intact EGCG was significantly higher for samples containing β-Lg-EGCG complexes. FRAP and ORAC assays also indicated that the antioxidant activity of β-Lg-EGCG complexes was significantly higher compared to free EGCG during intestinal digestion. These results support the hypothesis that binding of EGCG by heat induced β-Lg nanoaggregates protected EGCG from degradation and loss of its antioxidant activity during digestion. 

The in vitro bioaccessibility and in vivo bioavailability of EGCG in β-Lg-EGCG complexes was also determined in the study of Zagury et al.; the bioaccessibility was studied by analysing samples taken during in vitro gastric and intestinal digestion [87]. The samples were centrifuged through a membrane with a molecular weight cut-off of 3 kDa, and the released EGCG concentration was quantified using RP-HPLC/UV. The bioaccessibility was expressed as:

(1) $$\mathrm{bioaccessible}\;\mathrm{ECCG}\;=\;\frac{E_{f}}{E_{0}}\times 100$$

where *E_f_* represents the amount of free EGCG, measured with the RP-HPLC/UV at different points during the digestion and *E*_0_ represents the amount of EGCG before digestion. The bioaccessibility of free EGCG was higher during gastric digestion, but bound EGCG had a higher bioaccessibility during intestinal digestion. Overall, the in vivo results indicate that bound EGCG had a better bioavailability compared to free EGCG. The bioavailability was studied in a rat model and β-Lg-EGCG complexes, free EGCG, heat induced β-Lg nanoaggregates without EGCG and a blank were consumed by rats. After consumption, the EGCG content in the blood plasma of rats was analysed at different time intervals. It was shown that during the first 30 min of digestion, free EGCG resulted in the highest level of EGCG in the plasma. After the first 30 min, more EGCG was present in the plasma of rats fed β-Lg-EGCG complexes. Therefore, it can be concluded that heat induced β-Lg nanoaggregates increased the bioavailability of EGCG, mainly due to protection of degradation by β-Lg during digestion in the small intestine.

##### Effect of Heated β-Lactoglobulin on Health Benefits of EGCG

Wu et al. studied the antitumor activity of heat induced β-Lg-EGCG complexes [88]. For this study, the inhibitory effect of free EGCG and β-Lg-EGCG complexes on different types of human cancer cells, 22RV1 (prostate), 769-P (renal), A375 (melanocarcinoma), C33-A (cervical), CACO-2 (colon), HeLa (cervical), MSTO-211H (mesothelioma), NCI-N87 (gastric), SK-OV-3 (ovarian), SPC-A-1 (lung), TE-1 (oesophagus) and normal cells NIH3 (mouse embryo fibroblast cells), AML12 (alpha mouse liver cells), CCD-18CO (normal human colon cells) were studied. The inhibition of growth attributable to EGCG in normal cells was almost negligible, but significant in all cancer cells. The β-Lg-EGCG complexes were more effective in inhibiting growth compared to free EGCG, especially in A375 and TE-1 cells. In TE-1 cells there was more early apoptosis, whereas A375 showed more late apoptosis. 

EGCG potentially reduces cardiovascular disease, obesity and diabetes [94,95,96]. Because of this, Zagury et al. studied if β-Lg-EGCG complexes improved the prevention of metabolic syndrome effects in a high fat diet induced obesity mouse model [87]. The mice were split in two groups, one group had a normal diet (ND), and the other group had a high fat diet (HFD). These groups were again split into four groups, receiving β-Lg-EGCG complexes, EGCG with milk, EGCG with water or just water, creating a total of eight mice groups. The mice underwent different types of analyses: faecal lipid analysis, hepatic triglyceride analysis, body fat mass analysis, glucose tolerance and insulin tolerance. The bound EGCG did not reduce the body fat gained by the mice with a HFD. However, it did reduce the faecal lipid concentration, in both the mice with a HFD and ND, and the haptic triglyceride level in mice with a HFD. The glucose tolerance and insulin sensitivity were also greater in mice fed β-Lg-EGCG complexes. From this study, the authors concluded that heat induced β-Lg-EGCG complexes can improve the health effects of EGCG, due to delivery, and offer potential for use in preventive nutrition/medicine. 

### 3.3. Blood Serum Albumin

The presence of blood serum albumin (BSA) in milk is associated with transportation of serum albumin in the blood through the maternal epithelial cells to the milk [75]. BSA consists of 583 amino acids, has a molecular weight of 66 kDa, isoelectric point of 5.3 and denaturation temperature of 64 °C [22,75]. When quercetin dissolved in dimethyl sulfoxide was added to BSA, creating a solution with 10% (*w*/*w*) dimethyl sulfoxide, nanoparticles with a mean diameter of 9–12 nm were formed (Figure 4E) [35,36] and the binding of quercetin was studied with fluorescence spectrometry. An excitation wavelength of 280 nm was used, and the measurements were performed between an emission of 200–500 nm, at 27 °C. The K_b_ of BSA with quercetin was 7.34 × 10^4^ M^−1^ and gave positive values for both the ΔH° (5.88 kJ·mol^−1^) and ΔS° (112.80 kJ·mol^−1^), which indicated that the main type of interaction was hydrophobic [35]. In the study of Chanphai and Tajmir-Riahi, tea catechins (C, ECG and EGCG) were added to BSA and dissolved in a 10 mM Tris-HCl buffer of pH 7.2. Their binding was studied with UV-visible spectroscopy between 255 and 350 nm and it was found that the K_b_ decreased with an increase in temperature from 298.15 K to 318.15 K, and an increase in molecular weight of the catechin increased the binding constant. With the lowest recorded K_b_ of 1.64 × 10^5^ M^−1^ for catechin at 318.15 K and highest K_b_ of 3.2 × 10^5^ M^−1^ for epigallocatechin gallate at 289.15 K. Further thermodynamic analysis of the results gave negative ΔH° values, indicating the presence of hydrogen bonds, and positive ΔS° values, indicating the presence of hydrophobic interactions [89]. The study of Chanphai and Tajmir-Riahi also reported the EE of tea catechins, which was between 45 and 65%, and an increase of molecular weight of the catechin increased the EE [89]. 

In terms of functionality, a 2,2-diphenyl-1-picrylhydrazyl (DPPH) and ABTS study showed that binding of quercetin to BSA did not influence the antioxidant activity of quercetin [35,36]. Free quercetin mostly oxidises in the first 24 h. Quercetin in BSA complexes only oxidised for 70% after 216 h, which shows that BSA partly protects quercetin from oxidation [35]. This protection against oxidation also occurred in intestinal fluid, indicating protection during digestion [36].

## 4. Conclusions and Future Prospects

In summary, the caseins and whey proteins in milk both have the ability to interact with PP, with hydrophobic interactions being particularly important. Hydrogen bonds also play a role, especially with more polar PP, such as resveratrol and sesamol. There is considerable variation/choice in the size, structure, EE and functional properties of milk protein-PP complexes. These complexes are influenced by the type and concentration of PP, type and concentration of proteins and environmental (e.g., pH, temperature, salt concentration) and processing (e.g., choice of process operation) factors. The size of the milk protein-PP complexes varies: WPI > WPI with pectin > β-Lg (unheated) > CNP > CM, r-CM > β-Lg (heated) > β-CM > BSA. The smallest complexes were BSA with quercetin (9 nm), with the largest being WPI aggregates with PP rich juice (100 µm). A smaller particle size might be preferred in terms of colloidal stability. The lowest recorded EE was 18% for CMs with EGCG, and the highest was >99% for curcumin and quercetin-casein complexes. In most systems, an increase in the PP and a decrease in protein content led to a decrease in EE. This was due to saturation of the hydrophobic regions of protein upon PP binding. The studies that focused on the digestion of milk protein-PP complexes indicated that the PP in the complexes were less prone to degradation during digestion, which resulted in higher bioaccessibility and bioavailability of PP. Binding of PP to milk protein systems does not decrease the antioxidant activity of PP. Indeed, it can help to increase the overall antioxidant activity due to the ability of the milk proteins to solubilise PP in a polar system. The effect of protein-PP complexes on the antitumor activity is dependent on the type of PP, type of encapsulation system and the type of tumour cells.

At this time, only limited research has been completed on the behaviour of PP during the digestion of milk protein-PP complexes. More digestion studies are needed on a range of milk protein systems with the same PP profile in order to compare the protective effect of these milk protein systems. The influence of PP on the gut microbiota may have a major influence on the positive health effects of PP [6]. However, no literature has focused on the effect of milk protein-PP binding on the effect of PP on the gut microbiota. Research has demonstrated that the size and morphology of whey protein aggregates influences the digestion behaviour [37]. The influence of morphology and size of casein systems has not been studied, and the influence this could potentially have on PP bound to protein systems is also not understood.

Even though certain areas of milk protein-PP systems need to be studied in more detail, this review concludes that casein and whey proteins have great potential for developing food-grade delivery systems for PP.

## Data Availability

Not applicable.
